# Gastrointestinal microbial community changes in Atlantic cod (*Gadus morhua*) exposed to crude oil

**DOI:** 10.1186/s12866-018-1171-2

**Published:** 2018-04-02

**Authors:** Andrea Bagi, Even Sannes Riiser, Hilde Steine Molland, Bastiaan Star, Thomas H. A. Haverkamp, Magne Olav Sydnes, Daniela Maria Pampanin

**Affiliations:** 1 Department of Mathematics and Natural Sciences, University of Stavanger, N-4036 Stavanger, Norway; 2 Centre for Ecological and Evolutionary Synthesis, Department of Biosciences, University of Oslo, PO Box 1066, Blindern, N-0316 Oslo, Norway; 3 IRIS-Environment, International Research Institute of Stavanger, N-4070 Stavanger, Norway

**Keywords:** Intestinal microbiome, Atlantic cod, Oil exposure, Polycyclic aromatic hydrocarbons, Fish gut microbiome, *Deferribacterales*

## Abstract

**Background:**

The expansion of offshore oil exploration increases the risk of marine species being exposed to oil pollution in currently pristine areas. The adverse effects of oil exposure through toxic properties of polycyclic aromatic hydrocarbons (PAHs) have been well studied in Atlantic cod (*Gadus morhua*). Nevertheless, the fate of conjugated metabolites in the intestinal tract and their effect on the diversity of intestinal microbial community in fish is less understood. Here, we investigated the intestinal microbial community composition of Atlantic cod after 28 days of exposure to crude oil (concentration range 0.0–0.1 mg/L).

**Results:**

Analysis of PAH metabolites in bile samples confirmed that uptake and biotransformation of oil compounds occurred as a result of the exposure. Various evidence for altered microbial communities was found in fish exposed to high (0.1 mg/L) and medium (0.05 mg/L) concentrations of oil when compared to fish exposed to low oil concentration (0.01 mg/L) or no oil (control). First, altered banding patterns were observed on denaturing gradient gel electrophoresis for samples pooled from each treatment group. Secondly, based on 16S rRNA sequences, higher levels of oil exposure were associated with a loss of overall diversity of the gut microbial communities. Furthermore, 8 operational taxonomic units (OTUs) were found to have significantly different relative abundances in samples from fishes exposed to high and medium oil concentrations when compared to samples from the control group and low oil concentration. Among these, only one OTU, a *Deferribacterales*, had increased relative abundance in samples from fish exposed to high oil concentration.

**Conclusions:**

The results presented herein contribute to a better understanding of the effects of oil contamination on the gut microbial community changes in fish and highlight the importance of further studies into the area. Our findings suggest that increased relative abundance of bacteria belonging to the order *Deferribacterales* may be indicative of exposure to oil at concentrations higher than 0.05 mg/L.

**Electronic supplementary material:**

The online version of this article (10.1186/s12866-018-1171-2) contains supplementary material, which is available to authorized users.

## Background

Marine environments continue to be exposed to oil pollution events as oil and gas production activities continue to expand [1]. Accidental and operational releases of crude oil remain a threat to marine biota, including fish, therefore monitoring activities play an important role. Atlantic cod (*Gadus morhua*) is a commonly used fish species in environmental monitoring in the North Atlantic [2]. For this species, the adverse effects of oil exposure, in particular, the damage caused by petrogenic polycyclic aromatic hydrocarbons (PAHs) − a class of compounds found in crude oil and oil products − is well documented [3, 4]. In addition, several biomarkers of petrogenic PAH exposure are well-established for this species [3, 5]. PAHs found in crude oil are well-known carcinogens due to their ability to form DNA and protein adducts following their primary metabolism in the liver [6, 7]. Liver enzymes initiate the excretion process of PAHs by transforming them into more water soluble conjugated metabolites, which are sometimes more toxic than their corresponding parent PAHs [8, 9]. The conjugated metabolites are then excreted via the bile into the intestinal tract. The fate of conjugated metabolites in the intestinal tract and their effect on the gut microbiota is still under investigation. It is generally assumed that conjugated biliary PAH metabolites either (1) pass through the gut unchanged and are eliminated via feces, or (2) are hydrolyzed spontaneously, reabsorbed through the intestinal wall and transported to the liver via the portal vein (enterohepatic recirculation) [10]. Considering the metabolic diversity of the gut microbiota, conjugated metabolites could also undergo biotransformation as a result of bacterial activity in the gastrointestinal (GI) tract [11]. Moreover, there might be mechanisms that exacerbate the toxic effects of pollutants by for example reactivating conjugated biliary metabolites [12]. Studies on the potential interactions between biliary metabolites and gut microbiota in fish are scarce, and most microbiota studies have so far been carried out on mammals, e.g., mice and humans [13, 14]. Their results indicate that gut microbiota can indeed be actively involved in biotransformation of environmental pollutants. Such biotransformation may influence the detoxification process and alter the fate of xenobiotic compounds. At the same time, these pollutants can in return influence the composition of the gut microbiota.

The species composition, richness and diversity of the fish gut microbiome has been studied extensively in fish species that are interesting for the aquaculture industry mainly with the interest of improving fish health through enhanced feed. Commonly used methods include cultivation and/or molecular profiling with denaturing gradient gel electrophoresis (DGGE) [15]. Based on the increasing use of next-generation sequencing techniques a diverse gut microbiome composition has been detected in fish [16, 17]. While next-generation sequencing [18] indicated changes in the intestinal community of a freshwater fish (goldfish, *Carassius auratus*) upon exposure to an environmental pollutant (pentachlorophenol), studies investigating such effect in marine fish are lacking. Herein, we report the effect of oil exposure on the intestinal microflora in Atlantic cod. The aim of this study was to assess the potential shift in gut bacterial community composition of Atlantic cod due to oil exposure. The intestinal bacterial community composition, as analyzed by Illumina sequencing of V4 amplicons of bacterial 16S rRNA genes, was compared among gut microbiota of fish after a 28-day exposure to four levels of oil concentration (i.e., no oil, 0.01, 0.05 and 0.1 mg/L). Results from biological effect measurement, i.e. PAH metabolite analysis in fish bile, were used to confirm metabolic uptake of oil by the fish [2].

## Methods

### Exposure set up and sampling

Atlantic cod (weight range of 137–1455 g) were caught in March–April 2015 in the Stavanger region, transported to the International Research Institute of Stavanger facilities and placed in 1000 L tanks. After a 1–2 weeks’ acclimation, fish were exposed to three different concentrations of dispersed crude oil from the Troll C platform (North Sea, Norway): low (0.01 mg/L) (n of fish = 8), medium (0.05 mg/L) (n of fish = 9) and high (0.1 mg/L) (n of fish = 6). A group of fish were kept in a tank with clean seawater flow and used as control (n of fish = 7). A constant flow of 8 L/min seawater into each tank was ensured in both control and exposed groups. The crude oil was continuously distributed from a header tank (dispersed oil reservoir) into the different exposure tanks through a continuous flow system (CFS) as described earlier [19], in order to mimic natural environmental conditions, e.g., oil spill scenario . Illustration of the CFS system is shown in an additional figure [see Additional file 1]. The CFS was checked daily, oil droplet sizes were monitored three times a week by Multisizer measurements, and PAH concentrations were measured semi quantitatively by fixed wavelength fluorescence (FF) weekly (data not reported). A detailed description of the exposure set up can be found in the thesis of Delgado [20].

After 28 days of exposure, fish were sedated using 5 mg/L Aquacalm fish sedative (Metomidate HCl) and sacrificed by a sharp blow to the head. The entire intestinal tract was removed, placed in a sterile container, snap-frozen in liquid nitrogen and stored at − 80 °C until DNA extraction. For analysis of PAH uptake and biotransformation in fish, bile samples were drawn using a syringe, snap-frozen in liquid nitrogen and stored at − 80 °C until analysis.

### Support parameters

Chemical analysis was carried out in order to assess PAH content of the Troll C crude oil and of samples taken from the header tank on day 14 and 28 (Intertek West Lab AS, ISO28540:2011). In the Troll C crude oil, the EPA (Environmental Protection Agency) PAHs were quantified, while in the header tank, 25 PAH compounds and groups of compounds were measured.

Morphometric measurements, i.e., length, total weight of fish and liver weights, were recorded for all sampled specimens. General physiological indices were calculated as follows [21, 22]:

Condition Index (CI) = (weight (g)/[length (cm)]^3^) × 100.

Hepatosomatic Index (HSI) = [liver weight (g)/fish weight (g)] × 100.

PAH metabolites in bile were determined by two methods, the semi-quantitative fixed wavelength fluorescence (FF) and gas chromatography mass spectrometry (GC-MS).

Fixed wavelength fluorescence (FF) analysis of bile samples was conducted as described by Aas et al. [23]. Bile samples were diluted in 50% methanol and analysed using a Lumina fluorescence spectrometer (Thermo Scientific). The concentration of PAH metabolites was expressed as mg pyrene fluorescence equivalents (PFE)/mL bile.

GC-MS analysis was carried out as previously described [24, 25]. In brief, the OH-PAHs were extracted with ethylacetate, dried with anhydrous sodium sulphate and concentrated prior to sylilation with N,O-Bis(trimethylsilyl)trifluoroacetamide (BSFTA). Trimethylsilyl (TMS) ethers of OH-PAHs were analysed using an HP5890 series II gas chromatograph (Shimadzu QP2010) equipped with a CP-Sil 8 CB-MS (Varian) column. Mass spectra were obtained at 70 eV in selected ion mode (SIM).

### Gastrointestinal microbial community

#### DNA extraction

The GI tract of three fishes from each exposure group were used for microbial community composition analysis. After thawing on ice, each GI tract sample was cut into small pieces using sterile scissors and tweezer and placed in 15 mL sterile tubes with 3 mL ATL lysis buffer (Qiagen). Samples were vortexed vigorously several times and kept at room temperature (approx. 22 °C), then centrifuged briefly at 10,000 rpm. The liquid phase was decanted into a fresh 15 mL tube and contents were lysed overnight at 55 °C. Following this overnight incubation, a 30 min RNA digestion was carried out at 37 °C by adding 20 μL RNAse A solution (100 mg/mL, Qiagen) to each sample. DNA was then purified using phenol:chlorophorm:isoamylalcohol (PCI, 25:24:1, Sigma) extraction and ethanol precipitation. Briefly, to each lysate (approx. 3 mL), 5 mL of PCI was added and mixed vigorously. Samples were kept on ice followed by centrifugation at 5000 rpm (4 °C) for 15 min. The supernatant was pipetted into a new tube before the extraction was repeated one more time with PCI (5 mL) and then once with 5 mL chlorophorm:isoamylalcohol (CI, 24:1, Sigma). The DNA was finally precipitated with ice-cold ethanol (2 volumes of 100%) in the presence of sodium acetate (0.3 mM, pH 5.3). Samples were gently mixed and then incubated at − 20 °C for a minimum of 4 h. Falcon tubes were centrifuged at 5000 rpm for 30 min to collect the DNA pellet. Ethanol was discarded, a washing step with 70% ethanol was carried out and pellets were re-suspended in Tris-EDTA buffer (10 mM Tris-HCl, 1 mM EDTA, pH 7.8). Finally, an additional clean-up was performed using Genomic DNA Clean & Concentrator (Zymo Research) according to the manufacturer’s instructions, to completely remove inhibitors and resuspend DNA in molecular grade water.

#### PCR-DGGE

Polymerase chain reaction-denaturing gradient gel electrophoresis (PCR-DGGE) was carried out on all individual genomic DNA samples and on pooled genomic DNA samples which were from the same exposure group. The V3-V5 region of the 16S rDNA was amplified as described previously [26] (forward primer 341f: 5‘-CCTACGGGAGGCAGCAG-3’ with a GC-clamp 5′- CGCCCGGGGCGCGCCCCGGGCGGGGCGGGGGCACGGGGGG-3′ attached at the 5′-end and reverse primer 907r: 5′- CCGTCAATTCMTTTGAGTTT-3′). Each 50 μL PCR mix was composed of molecular grade water, 5 μL PCR buffer (5 Prime), 0.1 mM of each dNTP’s, 10 pM of each primer, 1 μL of genomic DNA and 0.25 μL of Taq polymerase (5 U/μL, 5 Prime). The PCR program was as follows: initial activation at 94 °C for 2 min, followed by 25 cycles of: (1) denaturation at 94 °C for 30 s, (2) annealing at 55 °C for 40 s and (3) elongation at 72 °C for 1 min. A final elongation step at 72 °C for 7 min was also included. The DGGE was carried out on a 6% acrylamide gel containing denaturing agents, urea and formamide, in a gradient of 20–80% prepared by using the IngenyPhorU gradient former. Equal amounts of PCR products were loaded into each well (confirmed by agarose gel electrophoresis). The run was performed using an IngenyPhorU (Ingeny International BV, Goes, The Netherlands) system (17 L volume, 1 x TAE as buffer, temperature at 60 °C) at 90 V for 18 h. Bands were visualized using post-staining (GelRed in TAE) for 60 min prior to imaging.

#### Amplicon sequencing and bioinformatics analysis

Amplicon libraries were prepared using BioScientific’s NEXTflex V4 library preparation kit following the manufacturer’s instructions with minor modifications. All DNA samples were diluted 10 times prior to first PCR step. Primer and barcode sequences are shown as additional file [see Additional file 2]. Instead of the recommended 10 cycles, 25 cycles were used during the first PCR reaction in order to obtain sufficient amount of products. PCR clean-up was then performed using MinElute Column Clean-up kit (Qiagen) and cleaned reaction mixes were diluted. The second PCR step was performed using 25 cycles as well. Approximately 40 μL of PCR products were loaded on a 2% agarose gel, bands corresponding to expected product size were excised and the second cleanup step was performed with MinElute Gel Extraction kit (Quiagen). DNA concentration was then measured with a NanoVue instrument (GE Healthcare). Products from three repeated library preparation rounds were pooled together prior to amplicon sequencing. Finally, the amplicons were sequenced by the Norwegian Sequencing Center (NSC) using Illumina MiSeq (Illumina, San Diego, CA, USA) V3 chemistry reagents (600 cycle, paired end). PhiX was blended in at 30% and a maximum of 1 bp mismatch was allowed during demultiplexing. All sequences have been deposited in the European Nucleotide Archive (ENA), acc. nr. PRJEB21667.

Read qualities were analysed using FastQC [27], primer and adapter sequences were removed using “cutadapt” [28] (standard parameters with primer sequences), before remaining phiX sequences were removed by mapping the reads to the phiX reference genome (Genbank acc. nr. J02482.1) using “BWA mem” with default settings [29]. Reads aligning were removed using seqtk with the *subseq* command [30]. Downstream processing and sequence analysis was performed using Mothur (v. 1.36.1) according to the Mothur Illumina MiSeq SOP [31]. Briefly, paired-end reads were joined using “make.contigs” and filtered based upon a minimum average Phred quality score of 25, contig length (max 289 bp) and a zero-tolerance to ambiguous base pairs. Chimeras were removed using the UCHIME algorithm [32] implemented in Mothur, before the sequences (V4 region) were aligned and classified using the Silva SEED bacterial database (v. 119) as reference. The dataset was clustered into OTUs based on a 97% similarity sequence similarity using average neighbour clustering. The OTU table output of Mothur was imported into R studio [33] (based on R (v. 3.2.3) [34]) for further processing using the R package “phyloseq” (v. 1.14.0) [35]. Results were visualized using the R package “ggplot2” (2.0.0) [36].

A common scaling procedure [37] normalized the OTU count in a given library with a factor corresponding to the ratio of the smallest library size in the dataset to the library size of the OTU in question. This replaces rarefying (i.e. random sub-sampling to the lowest number of reads) as it effectively results in the library scaling one would achieve by averaging an infinite number of repeated sub-samplings. We then excluded all OTUs present in only one sample from the dataset. Next, rarefaction curves were calculated on the observed number of reads to confirm a sufficient sequencing effort using an R script from the Phyloseq repository website [38]. Finally, alpha diversity measures and ordination distances for non-metric multidimensional scaling (NMDS) plots were calculated in Phyloseq. Distances were calculated based on OTUs with more than 10 reads (> 99% of total number of reads).

Differences in within-sample (fish specimen) diversity (alpha diversity) were tested using both analysis of variance (ANOVA) and the Wilcoxon rank-sum test. Further, differences in bacterial community composition (beta diversity) between the exposure groups were assessed using Permutational Multivariate Analysis of Variance (PERMANOVA) using the “adonis” function in the R package “Vegan” [39]. The test was run with 20,000 permutations, applying both OTU-based (Bray-Curtis/Jaccard) and phylogeny-based (weighted and unweighted UniFrac) measures. PERMANOVA was also applied to test for differences in PAH metabolites between the exposure groups. PERMANOVA assumes the multivariate dispersion in the compared groups to be homogeneous; this was verified using the “betadisper” function in “Vegan”. Similarity percentage (SIMPER) procedure implemented in “Vegan” was used to quantify the contribution of individual OTUs to the overall Bray-Curtis dissimilarity between the groups. “MetaStats” [40] was used to test for OTUs and orders with statistically significant differential abundances between the exposure groups. Only OTUs containing more than 0.1% of the total number of reads in the normalized and filtered dataset were used in this analysis. Finally, a correspondence analysis (CA) (“*cca*” in Vegan) was performed to explore the gut microbial community structure. Environmental parameters (order abundances) that significantly (*p* < 0.01) correlated with the ordination were fitted onto the CA plot using the “*envfit*” command in Vegan.

## Results

### Support parameters

Chemical analysis of the Troll C crude oil used in this exposure confirmed that all 16 EPA PAHs were present, their sum was 1.6 g/L. The most abundant PAHs present were 2- and 3-ring PAHs and the highest concentrations were found for those of 2-methylnaphthalene and 3-methylnaphthalene. The PAH profile of the seawater in the header tank also showed presence of both low and high molecular weight PAHs. Chemical analysis results for Troll C crude and header tank are summarized in additional files [see Additional files 3 and 4, respectively].

Morphometric parameters (CI and HSI) and concentrations of PAH metabolites in fish bile, reported for the three individuals used for GI microbial community analysis, were intended to give an overall description of the effect of oil on the exposed fishes. Moreover, concentrations of PAH metabolites in fish bile were used as support parameter for the GI microbial community analysis.

CI and HSI values, reported in additional tables [see Additional file 5], showed no differences between fish exposed to crude oil and the control group. The lack of significant difference between exposure groups was confirmed by statistical analysis using data from all exposed fishes (data not shown).

**Fig. 1:**
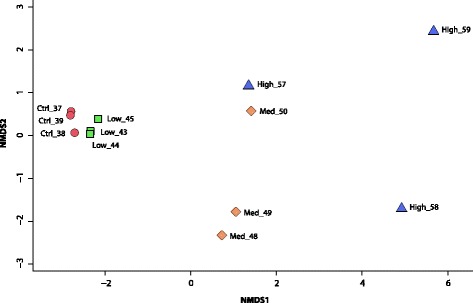
Differences in oil exposure levels in Atlantic cod. Oil exposure was assessed by multivariate analysis of PAH metabolite concentrations using non-metric multidimensional scaling (NMDS). The analysis is based on standardized polycyclic aromatic hydrocarbon (PAH) metabolite levels in fish bile, generated using Euclidean distances between samples. Fish subjected to the same oil exposure level are coloured identically. Ctrl = control, Low = low oil concentration, Med = medium oil concentration, High = high oil concentration. Numbers indicate sample (individual fish) numbers

The results of both FF and GC-MS analysis of PAH metabolites in bile confirmed the uptake and biotransformation of PAHs after exposure and results can be seen in additional files [see Additional files 6 and 7, respectively]. Distribution pattern of PAHs found in Troll C crude oil was reflected through the distribution pattern of PAH metabolites in the bile analysed by FF. Highest concentrations were measured for 2–3-ring PAH metabolites, while 4-ring and 5-ring PAH metabolites were present in lower concentration. GC-MS results further confirmed these observations, showing C_2_-OH-, and C_3_-OH-naphthalene to be found in the highest concentrations in bile from crude oil exposed fish. The level of PAH metabolites revealed a grouping of samples according to exposure level when using multi-variate analyses (Fig. 1). A non-metric multidimensional scaling (NMDS) plot showed a distinct clustering of control and low oil concentration exposed fish, clearly separated from the cluster of medium and high oil concentration exposed individuals.

Variation among individual fish was highest for the high exposure group as revealed by the scattering. The plot indicates a dose-response effect. Moreover, PERMANOVA analysis confirmed a significant difference between the control and low oil concentration exposed fish when compared to the medium and high oil concentration samples (*p* = 0.002, R2 = 0.59, Pseudo-F = 14.2, DF = 11) [see Additional file 8]. This, together with the NMDS plot, indicated a dichotomy between the lower (control + low) and upper (medium + high) exposure levels in the dataset.

### Gastrointestinal microbial community

**Fig. 2:**
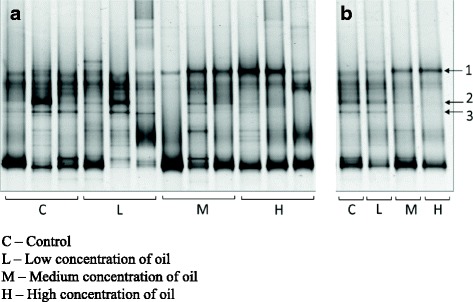
Denaturing gradient gel electrophoresis of 12 Atlantic cod gut bacterial communities from the four treatment groups. **a** Individual samples **b** Samples pooled from each exposure level

Three individual fish were randomly selected from each exposure group for DNA extraction and 16S rRNA gene-based community composition analysis. First, bacterial community compositions were compared by PCR-DGGE. This first screening showed that there were large variations among individuals as shown by discrete band patterns (Fig. 2a). However, some bands were clearly present in all samples. There was also clear distinction between the band patterns of the pooled samples of lower and upper oil exposure groups (Fig. 2b). One band (#1 on Fig. 2b) in particular appeared to be more dominant in the upper exposure group while two other bands (#2 and #3 on Fig. 2b) were less dominant in comparison to the lower exposure group.

**Fig. 3:**
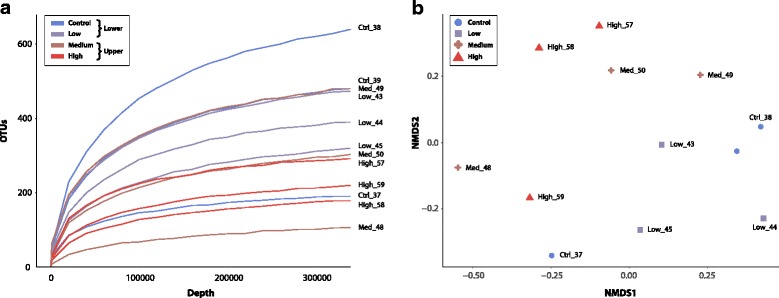
Diversity of microbial gut communities in Atlantic cod exposed to oil. **a** Rarefaction curves of 16S rDNA sequences at the 97% similarity cut-off. Specimens were exposed to increasing levels of oil exposure (*blue to red*). Curves are labelled with sample name (treatment level and fish specimen numbers). All samples are normalized to the same sequencing depth. **b** NMDS plot of all samples based on Bray-Curtis dissimilarity. Oil exposure treatment codes: Ctrl = Control, no oil, Low = low concentration of oil, Med = medium concentration of oil, High = high concentration of oil. Fish from the lower and upper exposure groups are colored in red and blue tones, respectively, and fish specimen numbers are shown after treatment codes

The paired-end sequencing of the 12 amplicon libraries resulted in a mean raw read number of 1,313,000 per sample [see Additional file 9]. After removal of phiX and other non-bacterial sequences we obtained a total of about 6 million assembled reads and a median of approx. 540,000 reads per sample [see Additional file 10]. After normalizing OTU abundances by common scaling based on the smallest library size (352,363 reads), and removal of OTUs present in only one sample, 994 OTUs representing > 99% of the reads in the dataset were identified. Rarefaction curve analyses on the normalized data, depicting the relationship between read number and number of detected OTUs, confirmed that the number of OTUs detected per sample was not caused by uneven sequencing depth (Fig. 3a). The difference in microbial communities between the lower and upper exposure groups was also suggested by their separation (blue vs. red tones) in an NMDS plot based on Bray-Curtis dissimilarity between the samples (Fig. 3b), as well as from the DGGE patterns observed for pooled samples (Fig. 2b).

The 12 GI microbial community samples contained between 133 and 720 OTUs and varied in diversity estimated by Shannon (H) and Inverse Simpson (1/D) indices (Table 1).

**Table 1:** Within-sample (alpha) diversity estimates of the intestinal microbiome in Atlantic cod specimens exposed to oil

Sample	Exposure level	Observed OTUs	Mean ± SD	Shannon	Mean ± SD	Inverse Simpson	Mean ± SD
37	Control	220	497 ± 254	1.30	1.88 ± 0.52	2.00	3.46 ± 1.58
38	Control	720		2.05		3.25	
39	Control	551		2.30		5.14	
43	Low	546	454 ± 84	1.99	2.1 ± 0.15	2.73	4.21 ± 1.28
44	Low	436		2.27		5.03	
45	Low	380		2.03		4.86	
48	Medium	133	342 ± 201	0.54	1.3 ± 0.73	1.31	2.15 ± 0.75
49	Medium	535		1.99		2.72	
50	Medium	358		1.36		2.42	
57	High	322	262 ± 56	1.60	1.45 ± 0.25	3.26	3.08 ± 0.63
58	High	212		1.16		2,38	
59	High	251		1.59		3,61	

All measures are based on normalized data. OTUs are clustered according to 97% sequence similarity cut-off value

While the number of observed OTUs appeared to decrease with increasing levels of oil exposure, no significant effect on the number of OTUs was determined by ANOVA (*p* = 0.37) as variation within treatment was high. Likewise, no significant differences in Shannon and Inverse Simpson diversity were detected between the four treatment levels in an ANOVA analysis. When comparing the treatment levels as partitioned into a lower (control + low) and an upper (medium + high) exposure group, however, a significant difference in Shannon diversity was identified using both ANOVA (*p* = 0.03, F = 6.04, DF = 1) and a Wilcoxon rank-sum test between the two groups (*p* = 0.04, W = 31). Moreover, PERMANOVA analysis of differences in community composition between the lower and upper exposure group was performed using four different beta diversity measures [see Additional file 8]. Significance values for Bray-Curtis dissimilarity and the Jaccard index were both less than 0.05, although not below the Bonferroni corrected *p*-value of 0.0125 (0.05/4). Nevertheless, this analysis also indicated a shift (decrease) in the overall intestinal microbial diversity between cod exposed to lower and upper levels of crude oil.

**Fig. 4:**
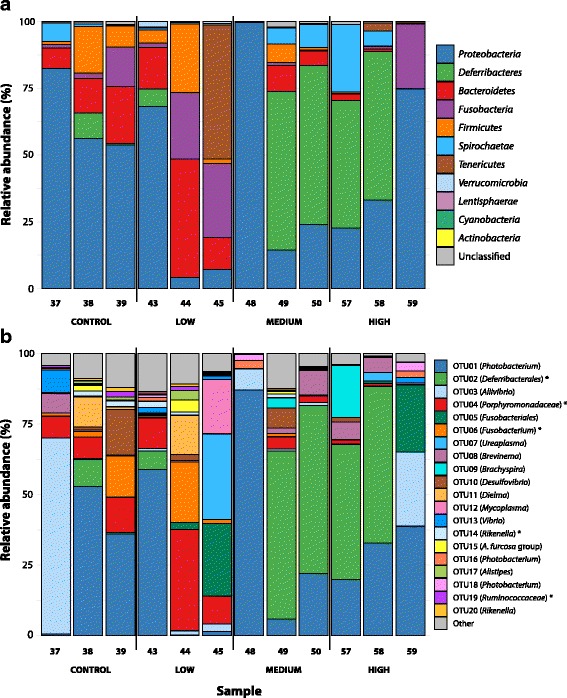
Taxonomic composition of the gut microbiome in Atlantic cod. Relative abundance of (**a**) bacterial phyla and (**b**) OTUs. Colors represent the 11 phyla (**a**) or the 20 most abundant OTUs (**b**) in cod. Unclassified sequences (**a**) and remaining OTUs (**b**) are merged into “Unclassified” and “Other” categories, respectively. Legend names are listed according to the total size of the taxonomic unit, and asterisks indicate OTUs differentially abundant between the lower and upper exposure groups

The 994 OTUs classified into 11 phyla with highly variable relative abundances among the cod individuals and treatment levels (Fig. 4a) [see Additional file 11]. A single abundant OTU was classified by a different method than the rest of the OTUs as described in an additional file [see Additional file 12]. The phylum *Proteobacteria* represented 45% (263 OTUs) of the sequences in the 12 cod samples, followed by the phyla *Deferribacteres* (20%, 1 OTU), *Bacteroidetes* (11%, 186 OTUs) and *Fusobacteria* (8%, 45 OTUs). *Proteobacteria* dominated 4 out of 6 GI communities in the lower exposure group and 2 out of 6 in the upper exposure group. The remaining two lower exposure samples, 44 and 45, were dominated by *Bacteroidetes* or *Tenericutes*, respectively. Phylum *Deferribacterales* had the largest relative abundance in the datasets obtained from the remaining 4 upper exposure samples (49, 50, 57 and 58). *Firmicutes* were present in relatively high abundance among lower exposure samples and in upper sample 49. *Fusobacteria* showed a similar trend with having high relative abundance in only one upper exposure sample, 59.

OTU level community compositions are shown in Fig. 4b. It is worth highlighting that a single OTU (OTU01), classified as a *Photobacterium* species, represented 30% of the total sequences in the 12 cod samples (Fig. 4b). This OTU represented 71% of all sequences classified as *Vibrionales*. OTUs assigned to the orders *Deferribacterales* (OTU02), *Vibrionales* (*Aliivibrio* species) (OTU03) and *Bacteriodales* (family *Porphyromonadaceae*) (OTU04) were also abundant.

The microbiome of the 12 sampled cod was further classified into 18 classes and 26 orders [see Additional file 13]. Further comparison of GI microbial community composition was performed on OTU and order level.

Eight OTUs had significantly different relative abundances between the lower and upper exposure groups according to MetaStats analysis (Table 2). This was in agreement with the SIMPER analysis, identifying these as some of the OTUs contributing the most to the observed (Bray-Curtis) dissimilarity between the two exposure groups [see Additional file 14]. Interestingly, the *Deferribacterales* OTU02 was the only OTU with a higher relative abundance in the upper exposure group. In contrast, OTU04, a *Bacteroidales* belonging to the family *Porphyromonadaceae*, was considerably more abundant in individuals from the lower exposure group.

**Table 2:** Differentially abundant (q < 0.05) OTUs in the intestinal microbiome of Atlantic cod specimens exposed to lower and upper levels of crude oil (see text for explanation)

	Lower		Upper				
	Mean	SE	Mean	SE	*p*-value	q-value	Taxonomy (genus)
OTU02	2.9%	1.8%	**38.2%**	12.2%	0.004	0.020	*Deferribacterales*
OTU04	**14.7%**	4.6%	1.6%	0.7%	0.005	0.020	*Porphyromonadaceae*
OTU14	**1.3%**	0.4%	0.3%	0.2%	0.017	0.029	*Rikenella*
OTU19	**0.8%**	0.3%	0.04%	0.01%	0.013	0.029	*Ruminococcaceae*
OTU20	**0.7%**	0.2%	0.11%	0.1%	0.014	0.029	*Rikenella*
OTU25	**0.4%**	0.2%	0.03%	0.02%	0.010	0.029	*Alistipes*
OTU28	**0.4%**	0.2%	0.03%	0.02%	0.025	0.037	*Clostridiales VadinBB60 group*
OTU29	**0.3%**	0.1%	0.04%	0.03%	0.005	0.020	*Clostridiales*

The 39 OTUs representing > 0.1% of the total abundance were analyzed using MetaStats. Bold values indicate the exposure level with highest mean abundance. SE = standard error

**Fig. 5:**
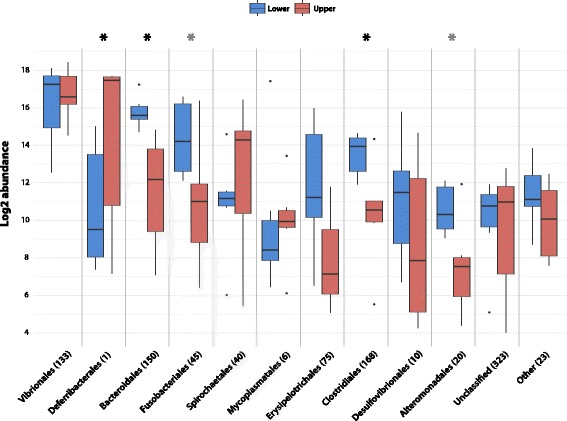
Log abundance of bacterial orders in two exposure groups of Atlantic cod. Black asterisks denote statistical significance as determined by MetaStats (*p* < 0.05). The grey asterisks represent orders that in a Wilcoxon rank-sum test have a *p*-value < 0.05 but is still above the Bonferroni corrected *p*-value (0.0045). Numbers in parentheses denote the number of OTUs in the corresponding order. All samples have been normalized to the same number of reads by common scaling

Normalized read counts (log2 transformed) of bacterial orders showed that in total five orders were differentially abundant in the two exposure groups (Fig. 5). Besides *Deferribacterales* and *Bacteroides* already mentioned above, *Fusobacteriales*, *Clostridiales* and *Alteromonadales* had a significantly lower abundance in the upper exposure groups.

**Fig. 6:**
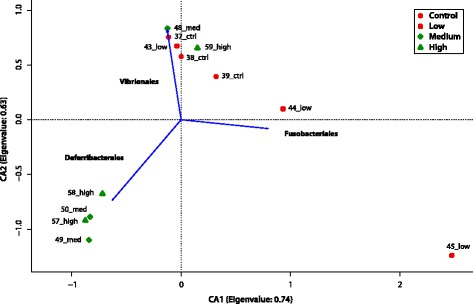
Divergence in microbial gut community structure in Atlantic cod exposed to oil. Correspondence analysis (CA) plot based on order level abundances. Eigenvalue for both axes are indicated in each axis label. Environmental parameters (orders) that significantly (*p* < 0.01) correlated with the ordination were fitted using the *envfit* command (Vegan package)

A correspondence analysis (CA) based on abundance of the different orders in the 12 cod individuals revealed a tightly clustered group of medium and high exposure samples (49, 50, 57 and 58) (Fig. 6). In concurrence with the findings from MetaStats analysis, fitting of environmental parameters (bacterial orders) demonstrated that these samples were significantly (p = 0.002) and positively correlated with *Deferribacterales* [see Additional file 15].

The other two samples from the upper exposure group (48 and 59) clustered together with control samples and the majority of low exposure samples. This was mostly due to the high abundance of *Vibrionales*. This order was indeed significantly correlated (*p* = 0.004) with these and most of the samples from the lower exposure group. There was also a significant correlation between abundance of *Fusobacteriales* and the lower exposure group samples (*p* = 0.005). Finally, samples 44 and 45 were positioned more peripherally on the CA plot driven by their abundances of *Bacteroidales* (phylum *Bacteroidetes*, sample 44) and *Mycoplasmatales* (phylum *Tenericutes*, sample 45), respectively.

## Discussion

Crude oil from the Troll platform, one of the largest oil fields on the Norwegian continental shelf, was used in order to investigate changes in GI microbial community composition of Atlantic cod upon a 28-day exposure, mimicking an environmental oil spill scenario. Chemical analysis showed that Troll C crude oil, similarly to other crude oils used in exposure studies of Atlantic cod, e.g., North Sea crude, contained significant amounts of PAHs.

Overall, this study showed that a 28-day exposure to Troll C crude oil at 0.01–0.1 mg/L resulted in no fish death and no significant changes in morphological parameters. This confirmed previous findings where no changes in CI and HSI were observed after 3-weeks exposure to produced water at 0.125% (total PAH 0.102 μg/L) in Atlantic cod [2]. Generally, a high CI and a high HSI indicate good health in fish and HSI in particular is considered the most sensitive growth indicator of fish. Nevertheless, CI can take up to several months prior to showing significant change due to environmental stressors [41]. Sub-lethal doses of crude oil were previously demonstrated to cause declining HSI, however, such effects are not always observed [2].

Following exposure to petrogenic PAHs, a wide range of conjugated oxidation products are accumulated in the bile of Atlantic cod [42, 43]. In this study, an increased level of PAH metabolites, showing a dose-response pattern, was found in bile samples of oil exposed fish. Bile metabolite measurement is considered as a sensitive tool for assessing exposure to oil and also produced water containing PAHs [6]. Several laboratory and field studies documented significantly elevated bile metabolite levels even at very low exposure concentrations similarly to the experiment presented here. PERMANOVA and NMDS analysis of GC-MS measured PAH metabolite concentrations in fish bile showed a dichotomy between the two lower (0.0 and 0.01 mg/L oil) and the two upper (0.05 and 0.1 mg/L oil) exposure levels. Based on these results, statistical analyses performed on the GI microbial community was done on pooled data, i.e., on data combined into lower and upper exposure groups.

OTU richness with numbers ranging from 133 to 720 OTUs identified in the 12 cod microbiomes were similar to that observed in analysis of 11 specimens of wild-caught Atlantic cod, where the number of OTUs per sample ranged from 40 to 228 [16]. A significantly higher number of OTUs (1850) were detected in 20 specimens of juvenile Atlantic salmon (salmon parr) at a similar sequencing depth [44]. Despite a relatively long time (28 days) spent under laboratory conditions, Shannon and Inverse Simpson indices varied greatly among individuals. Given the assumption that identical environmental conditions and feeding routines are expected to homogenize GI microbial communities this result was perhaps unexpected. Nevertheless, the ranges were somewhat narrower (Shannon: 0.54–2.30 and Inverse Simpson: 1.31–5.14) when compared to 11 specimens of wild-caught Atlantic cod kept under laboratory conditions for 12 days (Shannon: 0.30–3.07 and Inverse Simpson: 1.09–11.18). Large inter-individual variation in the intestinal microbial composition has been observed in other studies as well [44, 45] including GI microbial communities of a group of identically reared sibling zebrafishes [46]. Based on results emerging from next-generation sequencing studies of fish GI tract microbiota, the idea that each individual harbors a unique microbial ecosystem is being constantly reinforced [47]. Such high inter-individual variability has already raised the question of experimental reproducibility of exposure studies, which may be affected by the influence of highly variable fish associated microflora [48]. However, implications of this variability in terms of functional variability of the GI tract microbiome and host-microbiome interactions is yet unknown.

In the study presented here, the microbial contents of the entire GI tract were used from fishes that were starved 7 days prior to sampling. It is well known that different alimentary components harbor distinct microbial communities [47] and that starvation influences the composition of GI tract microbiota [49]. In future studies, it is preferable to collect intestinal contents based on a prior visual assessment of the alimentary anatomy.

On the phylum level, pentachlorophenol (PCP) exposure of goldfish for 28 days resulted mainly in increase in *Bacteroidetes* abundance and reduction of *Bacteroidetes*/*Firmicutes* ratio [18]. We did not observe such effect of oil on Atlantic cod in this study. Here, both *Bacteroidetes* and *Firmicutes* were present in lower relative abundances among upper exposure level individuals. Another recent study, which examined the effects of radio-labeled graphene nanoparticles of different size in adult zebrafish, also found that microbial communities were affected, and the size of the graphene nanoparticles seemed to be an important factor in determining the composition of GI tract microbiota [50]. While bacterial community composition of control fish (*n* = 4) and large nanoparticle exposed fish (n = 4) were similar, significant differences were observed in the gut microbiota composition of the small nanoparticle exposed fish (*n* = 4) based on NMDS analysis.

The intestinal bacterial community in our three control samples resembles that found in 11 cod individuals from the inner Oslo fjord [16], as it is dominated by *Vibrionales* and *Bacteroidales*, followed by *Clostridiales* and *Desulfovibrionales*. The large proportion of *Vibrionales* (median: 55%) in our samples is close to what is observed by Star et al. (median: 50%) [16] and is also in agreement with findings in other marine carnivores [51]. Further, members of the most abundant orders have previously been reported in Atlantic cod [49, 52–55]. Although the comparison with the Oslo fjord samples is based on a small sample sizes, the similarity between these communities indicates that our laboratory setting did not alter the species composition drastically compared to those found in wild populations.

*Deferribacterales* is a member of the recently discovered phylum Deferribacteres, and bacteria belonging to this group are considered acetoclastic iron-reducers. Iron is a nutrient that often limits bacterial growth, and animals typically try to reduce biologically available iron in order to avoid bacterial infections. Interestingly, it was found earlier, that exposure of juvenile Atlantic cod to HDF200 base oil (containing PAHs) for 30 days, resulted in downregulation of serotransferrin, a glycoprotein involved in iron-binding [56]. Lower levels of serotransferrin could result in higher levels of biologically available iron affecting microbial community composition in the cod gut.

The phylum *Deferribacteres* is present -albeit at low levels of relative abundance- in both the Oslo fjord cod [16] and our control and low-level individuals. Nonetheless, here we find that it is the sole order that significantly increased in relative abundance with increasing oil exposure levels. *Deferribacterales* has a wide environmental distribution and members have been found in hydrothermal vent communities [57], the gut microbiota of the hydrothermal shrimp *Rimicaris exoculate* [58] and in rodents [59]. Yet interestingly this order has also been found in high abundance in subsea petroleum reservoirs [60]. Moreover, it also occurs in relatively high abundance (19.2%) in biodegraded crude oil samples while it is absent in non-biodegraded samples from the same site [61]. This implies that *Deferribacteres* species thrive under oil exposed conditions. A recent metagenomic study of succession in a petroleum reservoir detected gene clusters encoding the pathway for anaerobic degradation of monoaromatic compounds in bins assigned to the genus *Flexistipes* (*Deferribacterales*) [62]. This could suggest that other genera in the *Deferribacterales* might be capable of breaking down aromatic hydrocarbons as well. However, since none of the described species within the *Deferribacteres* are implicated in the active breakdown of complex recalcitrant hydrocarbons [63], it is of interest to see the results of such experiments. Until then, we have to be cautious to interpret that our findings are due to catabolism of aromatic compounds by *Deferribacterales* species found in the Atlantic cod gut microbiome. Nonetheless, the *Deferribacterales* metabolize small molecules that are also abundant in the gut, such as monosaccharides, amino acids, short chain fatty acids in the presence of a suitable electron donor such as iron and nitrate. Therefore, when PAH exposure triggers higher iron concentrations in the cod gut, these bacteria could metabolize more of the small molecules and outcompete others. Our results provide another line of evidence suggesting that specific members of this order thrive in an environment exposed to oil, regardless of the presence of extant, complex microbial communities.

## Conclusions

It is recognized that the GI microbiome plays an essential role in gut health, immune function and nutrient uptake in fish. Although several studies have been investigating the intestinal microflora of various fish species, our current knowledge regarding the roles of gut microbiota in ecotoxicology remains limited. The exposure set-up in our experiment represents a realistic situation of chronic oil pollution. To the best of our knowledge, this is the first study demonstrating how exposure to sub-lethal concentrations of crude oil alters the intestinal microbiome of Atlantic cod. Despite the low number of samples, we identified 8 OTUs which were present in different relative abundances among lower and upper exposure groups. OTUs classified as *Porphyromonadaceae*, *Rikenella*, *Ruminococcaceae*, *Alistipes* and *Clostridiales* decreased in relative abundance. The only OTU which showed increased relative abundance at higher oil exposure was classified as *Deferribacterales*. Hence, *Deferribacterales* appear to be a potentially interesting microbial indicator of oil exposure in Atlantic cod. Further research with a larger sample number and functional analysis is necessary, in order to understand the implications of such shift and the potential changes of metabolic functions of intestinal microorganisms in response to environmental pollutants.

## Additional files

Additional file 1: **Figure S1.** Continuous flow system supplying dispersed crude oil into the exposure tanks at different levels. (PDF 167 kb)

Additional file 2: **Table S1.** Primer and barcode sequences. (XLSX 9 kb)

Additional file 3: **Table S2.** Composition of Troll C crude oil. (XLSX 12 kb)

Additional file 4: **Table S3.** Polycyclic aromatic hydrocarbon (PAH) content of oil dispersion in header tank. (XLSX 12 kb)

Additional file 5: **Table S4.** Condition indices (CI) and hepatosomatic indices (HSI) of oil exposed Atlantic cod specimens. (XLSX 9 kb)

Additional file 6: **Table S5.** Level of polycyclic aromatic hydrocarbon (PAH) metabolites in fish bile samples measured by fixed fluorescence (FF). (XLSX 8 kb)

Additional file 7: **Table S6.** Level of polycyclic aromatic hydrocarbon (PAH) metabolites in fish bile samples measured by gas chromatography mass spectrometry (GC-MS). (XLSX 13 kb)

Additional file 8: **Table S7.** Result of PERMANOVA analysis. (XLSX 10 kb)

Additional file 9: **Table S8.** Library sizes. (XLSX 9 kb)

Additional file 10: **Table S9.** Assembled reads. (XLSX 9 kb)

Additional file 11: **Table S10.** Taxa levels and abundances. (XLSX 25 kb)

Additional file 12: Supplementary text describing the classification method used for an unclassified highly abundant OTU and phylogenetic tree. (DOCX 380 kb)

Additional file 13: **Table S11.** OTU table for all samples. (XLSX 222 kb)

Additional file 14: **Table S12.** Result of SIMPER analysis. (XLSX 16 kb)

Additional file 15: **Table S13.** Result of *envfit* analysis. (XLSX 9 kb)

## Abbreviations

ANOVA: Analysis of variance
BSFTA: N, O-Bis(trimethylsilyl)trifluoroacetamide
CA: Correspondence analysis
CFS: Continuous flow system
CI: Chlorophorm:isoamylalcohol
CI: Condition index
DGGE: Denaturing gradient gel electrophoresis
DNA: Deoxyribonucleic acid
ENA: European Nucleotide Archive
EPA: Environmental Protection Agency
FF: Fixed wavelength fluorescence
GC-MS: Gas chromatography mass spectrometry
GI: Gastrointestinal
HSI: Hepatosomatic index
NMDS: Non-metric multidimensional scaling
OTU: Operational taxonomic unit
PAH: Polycyclic aromatic hydrocarbons
PCI: Phenol:chlorophorm:isoamylalcohol
PCP: Pentachlorophenol
PCR: Polymerase chain reaction
PERMANOVA: Permutational multivariate analysis of variance
PFE: Pyrene fluorescence equivalents
rRNA: Ribosomal Ribonucleic acid
SIM: Single ion mode
SIMPER: Similarity percentage
TMS: Trimethylsilyl
