# Supplementary figures and images for: Gastrointestinal microbial community changes in Atlantic cod (Gadus morhua) exposed to crude oil
Source: BMC Microbiol. 2018 Apr 2;18:25. doi: 10.1186/s12866-018-1171-2 (PMC5879832; doi:10.1186/s12866-018-1171-2)

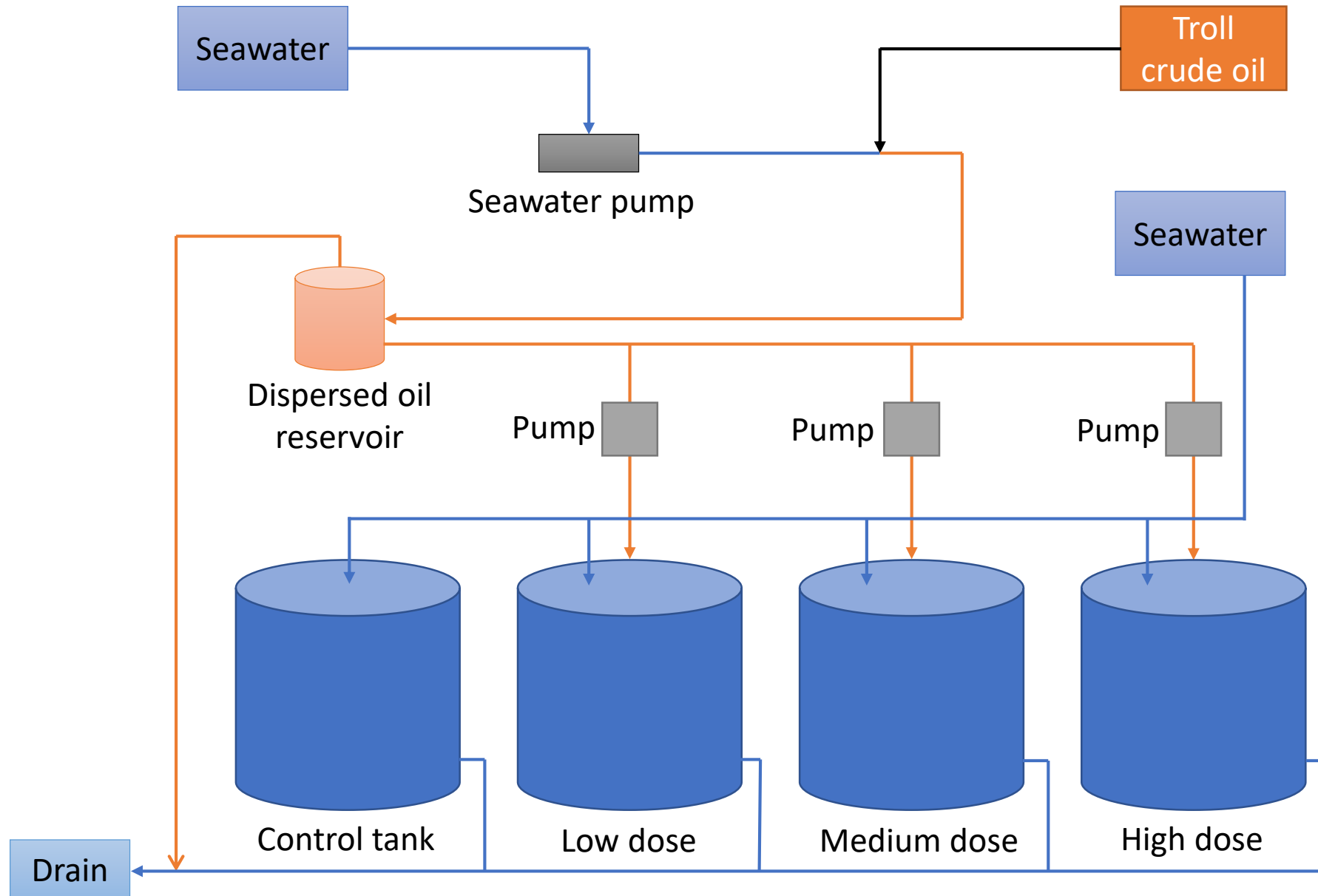

Supplement: Supplementary file 1 — Figure S1. Continuous flow system supplying dispersed crude oil into the exposure tanks at different levels. (PDF 167 kb) [file 12866_2018_1171_MOESM1_ESM.pdf]
