# Supplementary material for: Gastrointestinal microbial community changes in Atlantic cod (Gadus morhua) exposed to crude oil
Source: BMC Microbiol. 2018 Apr 2;18:25. doi: 10.1186/s12866-018-1171-2 (PMC5879832; doi:10.1186/s12866-018-1171-2)

**Phylogenetic classification of OTU00002**

The V4 sequence of OTU00002 was classified by alignment to the Silva SEED database (v. 119) as part of the Mothur amplicon pipeline. It’s classification, was «unclassified» at all levels, except «Bacteria». In order to obtain a better putative classification for OTU02, phylogenetic placement among closely related sequences was performed.

The OTU02 sequence was used to query the Silva SSU RefNR database (v. 128) using the SINA alignment service (v. 1.2.11, <https://www.arb-silva.de/aligner)> with the following settings: Minimum identity with query sequence: 0.7; 100 neighbors per query sequence. The top hit was acc. nr. JW184892 (Genbank classification: *Gadus morhua* mRNA), which had the Silva SSU classification: *Mucispirillum* sp. (order *Deferribacterales*). The remaining 99 hits included 16S rRNA sequences from the phyla: Deferribacteres (25) and Firmicutes (75), which is sister to the Deferribacteres when using the 16S rRNA gene. Due to the presence of many more Deferribacteres sequences in the database we collected sequences by downloading them from the Silva database using the phylum name for selection.

A reference dataset to align OTU02 sequences was created in the following way:

All available *Deferribacterales* sequences (83) were extracted from the SILVA SSU RefNR database with the following parameters: sequence length >1300, sequence quality: > 90, pintail quality: > 90. 16S rRNA Sequences of Firmicutes were retrieved with the same settings as for the Deferribacteres, except the SILVA living Tree database was used instead of the SILVA SSU refNR database, in order to reduce the number of sequences used (>1200). The Firmicutes dataset was further downsized by only selecting one sequence at the taxonomic family level (37 sequences).

For all Deferribacteres sequence we determined the isolation source by checking the genbank files for each sequence manually. This info was added to the header of each sequence. For the species *Deferribacter desulfuricans* SSM1 only one sequence was kept.

All full-length RNA sequences were imported to the Geneious software (v. 10.1.2, Biomatters Ltd., Auckland, NZ). Since our dataset did not include the sequence JW184892, we added it to the complete dataset. Similarly we added the OTU00002 sequence. Inside geneious we used Muscle with standard settings to align the complete dataset (121 sequences).

The V4 16S rRNA alignment was then manually trimmed and corrected to only contain the V4 region of the 16S and converted into DNA sequences for further processing. The resulting alignment was exported to MEGA version 7.0.20 and used for phylogenetic tree construction alignment. A Maximum Likelihood phylogeny was build using the GTR+G+I model (selected after modeltesting) with 500 bootstraps. The phylogeny was constructed using 121 16S rRNA sequences (262nt), using Firmicute sequences as an outgroup. Positions with gaps were deleted. In addition, a phylogeny was constructed using the Neighbor-Joining method with the Jukes-Cantor algorithm and 500 bootstrap replications. Both phylogenies showed OTU02 clustering closely with 16S rRNA sequences from the genus *Mucispirillum*. The resulting tree is shown below. OTU02 is indicated in green and with a star. Bacterial sequences associated with animals are indicated in red and a dot.


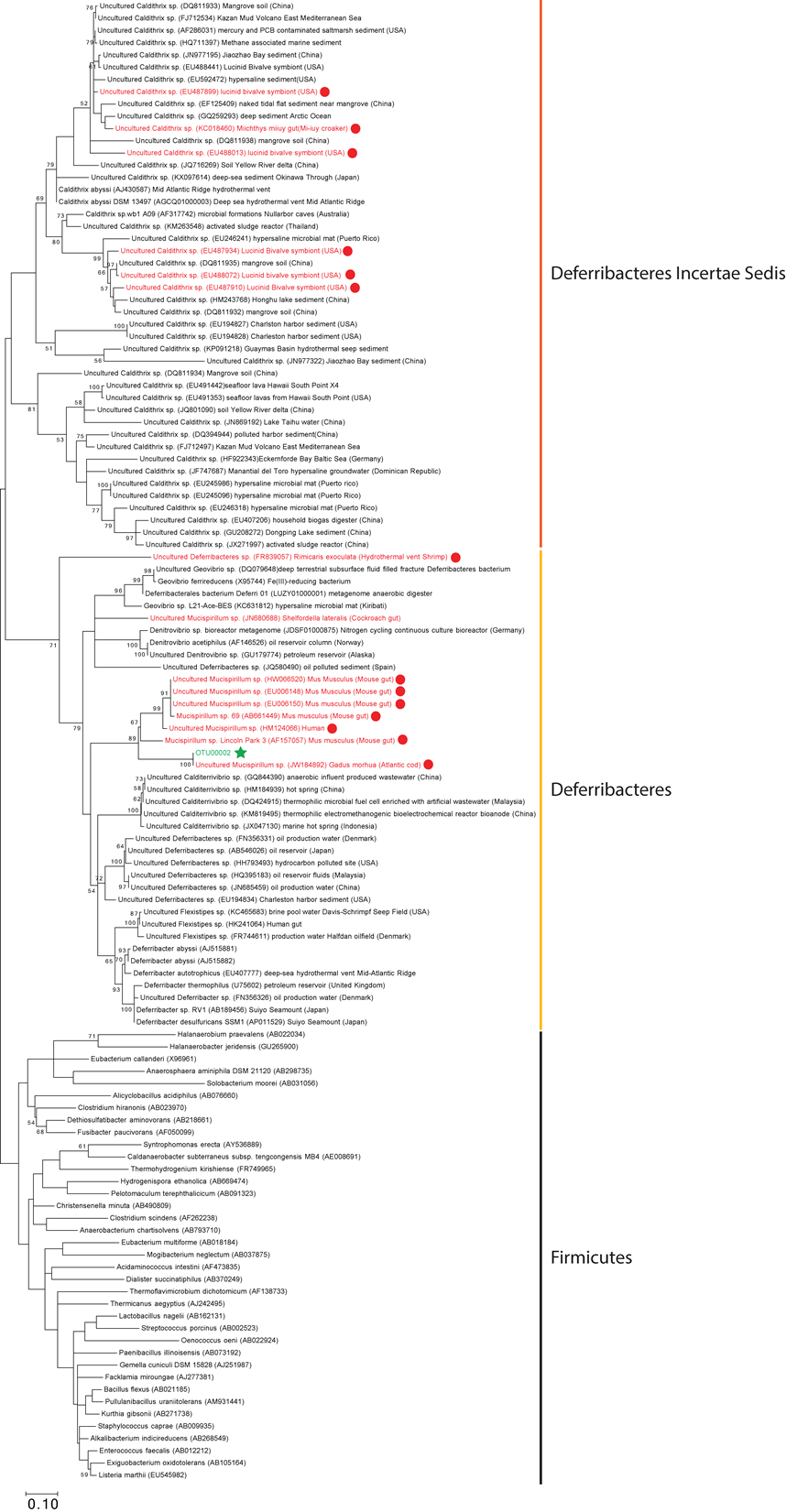

Supplement: Supplementary file 12 — Supplementary text describing the classification method used for an unclassified highly abundant OTU and phylogenetic tree. (DOCX 380 kb) [file 12866_2018_1171_MOESM12_ESM.docx]
